# Pityriasis Rosea

**Published:** 1908-06-06

**Authors:** 


### Dermatology.

### PITYRIASIS ROSEA.

Pityriasis Rosea is a more common affection than is generally supposed. It is, no doubt, sometimes disregarded by the patient because it occurs on covered parts, and gives rise to little or no subjective sensations. Frequently, too, it is mistaken for other affections—seborrhœic dermatitis, psoriasis, ringworm, or perhaps most often for a syphilitic eruption. Actually its characters are very distinct and constant. Often it occurs in what appear to be epidemics, and when one case has been seen others follow. It is said to be more common in the early spring. The eruption consists of rounded or oval patches or macules, of pale red colour, with little or no infiltration, hardly raised above the surface, and covered with small adherent scales.

The patches vary in size from a few lines to an inch or more across. They are generally distributed thickly over the trunk (back and front), and for a short distance along the upper arms and the thighs and on the neck. At the sides of the trunk the oval patches are arranged with their long axis obliquely downwards and forwards. This distribution on the trunk and stopping short just on the neck and limbs is one of the characteristic features of the eruption, and it helps one considerably in recognising the disease. In children, however, the eruption may sometimes be more limited in extent, while on the other hand it may extend beyond the usual area of distribution on to the face and scalp. Sometimes the patches tend to fade in the centre, and thus to produce circinate lesions. There is generally found one patch larger than the rest, which one is told was present for some days before the main eruption appeared. It has been termed the "herald patch." The eruption usually remains for three or four weeks, and then fades spontaneously; but it may persist for much longer periods. There may be premonitory symptoms, such as sore-throat and slight fever, but generally there are none.

From its sudden eruption and more or less definite course, and from the occurrence of epidemics, this affection has been likened to an exanthem. Some have supposed that local infection takes place at the seat of the "herald patch," and that this is followed by general infection. But there is no direct evidence of the presence of a micro-organism.

The importance of this eruption in practice lies mainly in its diagnosis, and especially in its diagnosis from ringworm of the body and from syphilis. It may also be confused with seborrhœa corporis, and with psoriasis.

In ringworm of the body the patches are never so numerous nor so regularly distributed over the characteristic seats of pityriasis rosea. The individual lesions are more sharply margined and more markedly ringed, while there are small papules or vesicles, or there is marked scaling, at the margin. In doubtful cases the diagnosis is settled by finding fungus in the scales from the ringworm lesions. In syphilis, except in the case of the early roseola, the patches are either infiltrated or they are markedly scaly, and their colour is deeper red or coppery. The early roseola, which has a somewhat similar distribution to that of pityriasis rosea, shows no scales, and, moreover, papular syphilitic lesions are often present at the same time. The diagnosis is confirmed by other evidences of early syphilis, such as the primary chancres or the sore-throat; but it must be remembered that the throat may be congested, and the cervical glands more or less enlarged in pityriasis rosea.

In seborrhœa the lesions are more infiltrated and the scales are thicker and greasy. Outlying follicular papules, which form the primary elements of the lesions, can also be made out on careful inspection. The scalp is affected with seborrhœa sicca, often also the eyebrows, and the flexures may be involved. In psoriasis the scales are very much thicker, and in large silvery flakes; the patches are of a deeper red.

The course of the eruption may be shortened by frequent soap and water baths, followed by inunction of a mild sulphur ointment or of an ointment containing salicylic acid (gr. x-xv. ad ℥j). If there is pruritus (and occasionally the eruption itches to some extent) an antipruritic lotion may be used in place of an ointment.

